# Different Patterns of Expression and of IL-10 Modulation of Inflammatory Mediators from Macrophages of Lyme Disease-Resistant and -Susceptible Mice

**DOI:** 10.1371/journal.pone.0043860

**Published:** 2012-09-14

**Authors:** Aarti Gautam, Saurabh Dixit, Monica Embers, Rajeev Gautam, Mario T. Philipp, Shree R. Singh, Lisa Morici, Vida A. Dennis

**Affiliations:** 1 Division of Bacteriology and Parasitology, Tulane National Primate Research Center, Tulane University Health Sciences Center, Covington, Louisiana, United States of America; 2 Division of Microbiology, Tulane National Primate Research Center, Tulane University Health Sciences Center, Covington, Louisiana, United States of America; 3 Center for Nanobiotechnology Research, Alabama State University, Montgomery, Alabama, United States of America; 4 Department of Microbiology and Immunology, Tulane University, Tulane University Health Sciences Center, New Orleans, Louisiana, United States of America; University of Kentucky College of Medicine, United States of America

## Abstract

C57BL/6J (C57) mice develop mild arthritis (Lyme disease-resistant) whereas C3H/HeN (C3H) mice develop severe arthritis (Lyme disease-susceptible) after infection with the spirochete *Borrelia burgdorferi*. We hypothesized that susceptibility and resistance to Lyme disease, as modeled in mice, is associated with early induction and regulation of inflammatory mediators by innate immune cells after their exposure to live *B. burgdorferi* spirochetes. Here, we employed multiplex ELISA and qRT-PCR to investigate quantitative differences in the levels of cytokines and chemokines produced by bone marrow-derived macrophages from C57 and C3H mice after these cells were exposed *ex vivo* to live spirochetes or spirochetal lipoprotein. Upon stimulation, the production of both cytokines and chemokines was up-regulated in macrophages from both mouse strains. Interestingly, however, our results uncovered two distinct patterns of spirochete- and lipoprotein-inducible inflammatory mediators displayed by mouse macrophages, such that the magnitude of the chemokine up-regulation was larger in C57 cells than it was in C3H cells, for most chemokines. Conversely, cytokine up-regulation was more intense in C3H cells. Gene transcript analyses showed that the displayed patterns of inflammatory mediators were associated with a TLR2/TLR1 transcript imbalance: C3H macrophages expressed higher TLR2 transcript levels as compared to those expressed by C57 macrophages. Exogenous IL-10 dampened production of inflammatory mediators, especially those elicited by lipoprotein stimulation. Neutralization of endogenously produced IL-10 increased production of inflammatory mediators, notably by macrophages of C57 mice, which also displayed more IL-10 than C3H macrophages. The distinct patterns of pro-inflammatory mediator production, along with TLR2/TLR1 expression, and regulation in macrophages from Lyme disease-resistant and -susceptible mice suggests itself as a blueprint to further investigate differential pathogenesis of Lyme disease.

## Introduction

Lyme disease, caused by infection with the spirochete *Borrelia burgdorferi*, is the most frequently reported vector-borne disease in the United States. Following infection, spirochetes disperse to multiple organs and persist in them for a long time causing many of the diverse manifestations of Lyme disease such as acute or chronic arthritis, carditis, and neuroborreliosis [1], [2]. Persistent tissue infections with *B. burgdorferi* often trigger immune responses directed against the spirochete and/or its lipoproteins [3], [4], [5], [6], [7], [8], [9], [10], [11], [12]. The potent stimulatory properties of the spirochete and its lipoproteins are thought to be responsible for inflammatory foci in tissues, as well as other host responses against the infection, all of which have been linked to Lyme disease pathogenesis [3], [5], [13], [14], [15].

The mouse model provides a unique opportunity to study the pathogenesis of Lyme disease, since experimental infection of different mouse strains with *B. burgdorferi* results in distinct disease outcomes that bear similarities to the different Lyme disease manifestations seen in humans [16], [17]. For example, C3H/HeN (or C3H/HeJ) mice develop severe arthritis (Lyme disease-susceptible) whereas C57BL/6J mice develop mild arthritis (Lyme disease-resistant) after infection with *B. burgdorferi* [18]. To understand these disease differences researchers have focused on cytokines and chemokines because these mediators regulate early innate immune responses, link innate and adaptive immunity, and are thought to play key roles in the pathogenesis of Lyme disease. Differences in their levels and patterns of up and down-regulation may be a decisive factor in survival of the spirochete during early infection and subsequent disease progression.

An earlier report showed that macrophages from disease-susceptible C3H mice that were stimulated with *B. burgdorferi* lipoproteins produced higher amounts of the prototypic IL-6 and TNF cytokines than did macrophages from disease-resistant C57 mice [19]. We similarly reported that lymph node cells from *B. burgdorferi*-infected C3H mice produced more of the inflammatory cytokines IFN-γ and IL-6 than did those of C57-infected mice, in response to lipoprotein stimulation [20]. Subsequent studies further revealed that expression of the chemoattractants CXCL1/KC and CCL2/MCP-1 correlated with Lyme arthritis based on their high expression levels in joints of *B. burgdorferi*-infected C3H mice as compared with joints of C57 mice [21], [22]. These early initial observations highlighted the potential roles that cytokines and chemokines may play in susceptibility and resistance to Lyme disease in the mouse model.

Inflammatory mediators that are induced by *B. burgdorferi* (or its lipoproteins) in cells from mice of Lyme disease-resistant and -susceptible strains have been shown to be tightly regulated by the anti-inflammatory cytokine IL-10. *In vitro* studies showed that IL-10 down- regulated the production of lipoprotein-induced IL-6 and TNF in macrophages of C57 and C3H mice [19]. Addition of exogenous IL-10 to lipopeptide-stimulated lymph-node cell cultures also reduced IFN-γ and IL-6 production with the inhibition being more effective with cells from disease-resistant C57 mice than with cells from disease-susceptible C3H mice [23]. Studies by Brown and coworkers [19] revealed that lipoprotein-stimulated macrophages of C57 mice produced higher levels of the anti-inflammatory cytokine IL-10 as compared to C3H mice. *In vivo* studies using IL-10-/- mice of both C57 and C3H genetic backgrounds underscored the significance of IL-10 in controlling joint inflammation in Lyme disease [19], [22]. Other investigators have also shown that several cytokine and chemokine genes are up regulated in joints of *B. burgdorferi*-infected IL-10 deficient mice [24], [25] indicating that the expression of these genes is tightly controlled by IL-10. These results give credence to the notion that IL-10 plays a pivotal role in Lyme disease pathogenesis in the mouse model.

The polarized Lyme disease outcomes in the resistant and susceptible mouse strains may ultimately depend on the expression and regulation of cytokines and chemokines as induced by spirochetes early after infection. However, comparative analyses of inflammatory mediators produced by macrophages from the C57 and C3H mouse strains have been partial, limited, and focused on a few mediators. These types of analyses only provide a limited scenario of what may actually occur after early cellular exposure to live spirochetes in Lyme-disease resistant and -susceptible mouse strains. The goal of the experiments presented herein is to provide a comprehensive analysis of the production of cytokines and chemokines by macrophages from C57 and C3H mice after these cells were exposed *ex vivo* to live *B. burgdorferi* spirochetes and a lipoprotein, and to evaluate how these mediators are regulated by IL-10. Our hypothesis is that susceptibility and resistance to Lyme disease, as modeled in mice, is associated with early induction and regulation of inflammatory mediators by innate immune cells after exposure to live *B. burgdorferi* spirochetes and/or its lipoproteins. We first assessed production/transcription of cytokines and chemokines in response to stimulation with both live *B. burgdorferi* and the lipoprotein outer surface protein A (L-OspA) using multiplex ELISA and qRT-PCR. Next we used qRT-PCR to assess transcriptional expression of genes encoding mediators of the TLR pathway after exposure of macrophages to these same stimuli. Finally, using the above experimental design, we assessed how the production levels of inflammatory mediators change in the presence of exogenous, or absence of endogenous IL-10. Our data suggest that the balance between Lyme disease-resistance and susceptibility correlates with, and may depend upon, a full pattern of expression of inflammatory mediators, and on the host's genetic ability to regulate such pattern, with IL-10 being a key mediator of this process.

## Results

### Bone marrow-derived macrophages from Lyme-disease resistant and disease-susceptible mice display, respectively, two distinct patterns of inflammatory mediator production

In the present study we employed multiplex and qRT-PCR assays to investigate quantitative differences in the expression levels of cytokines and chemokines produced by bone marrow-derived macrophages (BMDM) from C57 and C3H mice. BMDM were exposed *ex vivo* to live *B. burgdorferi* spirochetes (live Bb) or to the spirochetal lipoprotein, L-OspA. Upon stimulation, the production of both cytokines and chemokines was up-regulated in BMDM from both mouse strains. Interestingly, however, the magnitude of the chemokine up-regulation was larger in C57 BMDM than it was in C3H BMDM, for most chemokines. Conversely, cytokine up-regulation was more intense in C3H BMDM.

**Figure 1:**
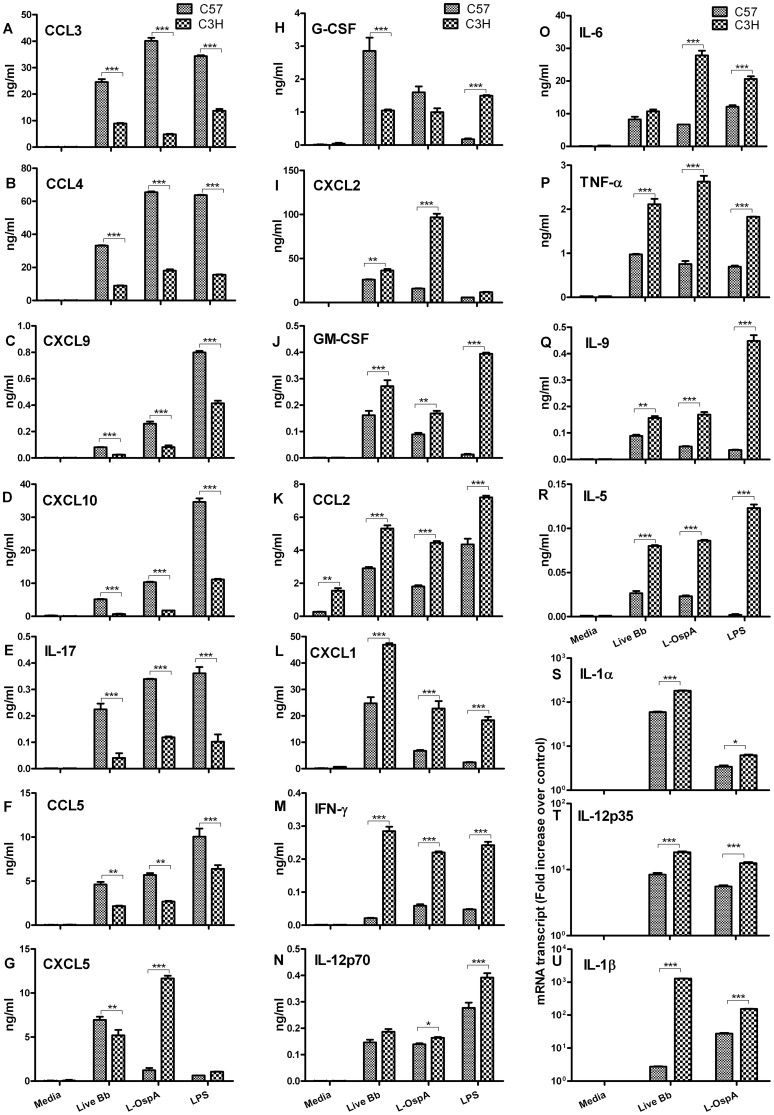
Distinct expression profiles of inflammatory mediators as produced by macrophages of Lyme disease-resistant and -susceptible mice. Bone marrow-derived macrophages (BMDM) at 0.5×10^6^/well from C3H and C57 mice were incubated with live *B. burgdorferi* spirochetes (live Bb) at an MOI of 10 or with lipidated outer surface membrane protein (L-OspA) at a concentration of 1 µg/mL. The control culture consisted of cells incubated with media alone without stimulants (Media) while lipopolysaccharide (LPS) at a concentration of 1 µg/mL was used as positive control for macrophage stimulation. (A–U) Cell-free supernatants collected from unstimulated and stimulated cultures at 24 h post-incubation were used to detect cytokines and chemokines using Milliplex ELISA detection system. (S–U) SABiosciences RT^2^ Profiler™ PCR Array was used to quantify mRNA transcript levels. Gene expression was normalized to internal controls (housekeeping genes) to determine the fold change in gene expression between test and control samples by the ΔΔCT method. Results are presented as fold increase over control (the level in unstimulated cells). A two-way Analysis of Variance (ANOVA) followed by a Bonferroni post-hoc test (GraphPad Prism 5) was used for data analyses. Significance was established at *P*<0.001 = ***, *P*<0.01 = ** and *P*<0.05 = *. Each bar represents the mean ± standard deviation for duplicate cultures. These experiments were repeated two times, with similar results.

The stimulatory patterns revealing quantitative differences in specific cytokine and chemokine expression levels between mouse strains are shown in **Fig. 1A–U**. C57 BMDM produced a pattern of inflammatory mediators characterized by significantly (*P*≤0.05) more elevated expression of a large number of chemokines (CCL3, CCL4, CCL5, CXCL9 and CXCL10) and fewer cytokines (IL-17 and G-CSF) (**Fig. 1A–F** and **Fig. 1H**) compared to those produced by C3H BMDM. In contrast C3H BMDM produced a pattern of inflammatory mediators characterized by significantly (*P*≤0.05) more elevated expression of a large number of cytokines (IL-1α, IL-1β, IL-5, IL-6, IL-9, IL-12p35, IL-12p70 TNF-α, IFN-γ and GM-CSF) and a much smaller number of chemokines (CXCL1 and CCL2) (**Fig. 1J–U**) compared to those produced by C57 BMDM. The transcriptional expression of IL-1α, IL-1β and IL-12p35 was evaluated by qRT-PCR (**Fig. 1S–U**) as their protein levels were too low for multiplex detection. The chemokines, CXCL2 and CXCL5 were not assigned to a mouse strain pattern because their levels as produced by macrophages of C57 and C3H mice, in response to live Bb or L-OspA stimulation were different between experiments (**Fig. 1G and I**). Similar patterns of inflammatory mediators were produced when lipopolysaccharide (LPS) was used as a stimulant. The data demonstrates two distinct patterns of inflammatory mediators displayed by macrophages from Lyme disease-resistant C57 and disease-susceptible C3H mice.

### IL-10 is more highly expressed by macrophages of the Lyme disease-resistant mouse than by those of the disease-susceptible mouse

**Figure 2:**
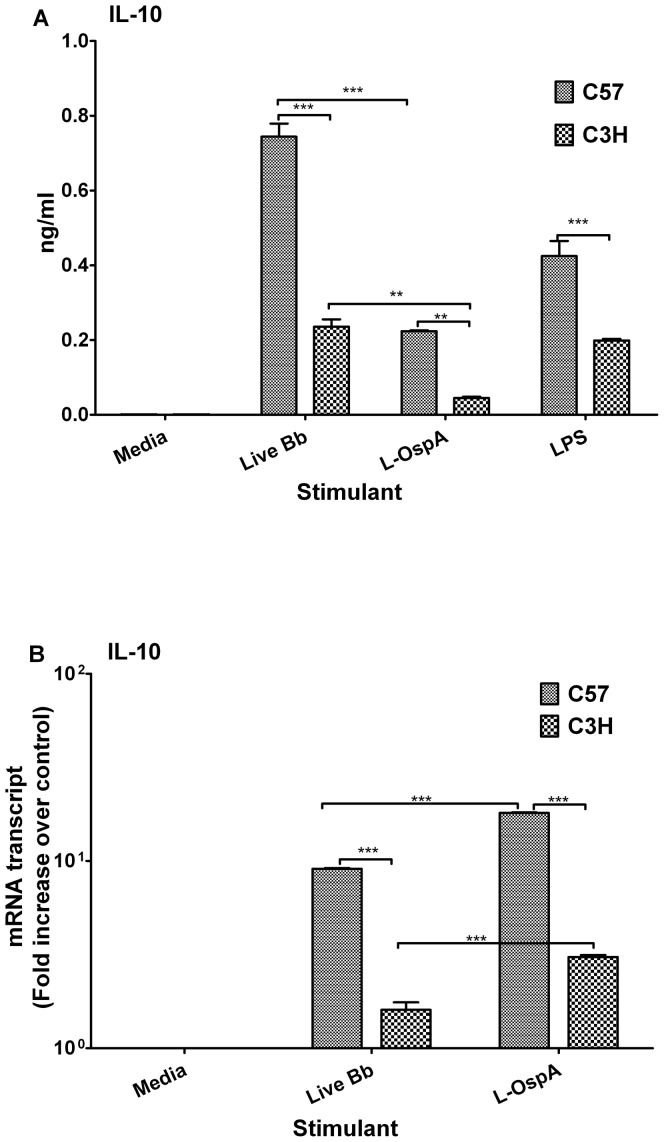
IL-10 is more highly expressed by macrophages of the Lyme disease-resistant mouse than by those of the disease-susceptible mouse. BMDM cultures were established as indicated in Fig. 1 and cell-free culture supernatants and RNA samples were collected after 24 h of incubation. (A) IL-10 protein concentrations were quantified using ELISA. (B) IL-10 mRNA gene transcripts were quantified by SABiosciences RT^2^ Profiler™ PCR Array as indicated in Fig. 1B. Results are presented as fold increase over control (the level in unstimulated cells). Data were analyzed using a one- or two-way ANOVA followed by a Bonferroni post-hoc test (GraphPad Prism 5). Significance was established at *P*<0.001 = ***, *P*<0.01 = ** and *P*<0.05 = *. Each bar represents the mean ± standard deviation for duplicate cultures. Data are representative of two independent experiments, with similar results.

Given the ability of the anti-inflammatory cytokine IL-10 to modulate inflammatory mediators in mouse macrophages in response to *B. burgdorferi* stimulants [19], [26], [27] we next measured IL-10 expression in BMDM of C57 and C3H mice to determine if IL-10 expression levels could provide some insights into their respective patterns of displayed inflammatory mediators. IL-10 protein and mRNA gene-transcript expression levels were significantly higher (*P*≤0.05) in live Bb- and L-OspA-stimulated C57 BMDM than they were in BMDM of C3H (**Fig. 2A–B**). In addition, live Bb induced significantly more IL-10 protein (*P*≤0.05) than did L-OspA (**Fig. 2A**) whereas L-OspA induced significantly higher IL-10 mRNA gene transcripts (*P*<0.001) than did live Bb (**Fig. 2B**). These findings suggest differential induction of IL-10 protein and mRNA transcript expression levels by live Bb and L-OspA. Thus as previously reported for OspA-stimulated C57 BMDM [19] our results show that C57 BMDM expressed more of the IL-10 protein but, as now reported, also its transcripts, in response to both live Bb and L-OspA stimulation. LPS-stimulated C57 BMDM also produced more IL-10 protein than similarly stimulated C3H BMDM (**Fig. 2A**). These studies suggest that IL-10, because of its anti-inflammatory properties, could potentially impact the levels of inflammatory mediators produced by macrophages, particularly in the C57 Lyme disease-resistant mouse strain.

### Transcription of genes related to TLR signaling is differentially induced by live spirochetes and lipoprotein in macrophages of C57 and C3H mice

Stimulation of inflammatory mediators by *B. burgdorferi* and its lipoproteins is mostly mediated via TLR signaling [5], [7], [11], [14]. Therefore we investigated how transcriptional activation of genes encoding mediators of the TLR pathway by live spirochetes and lipoprotein in BMDM may help explain their mouse-strain dependent patterns of inflammatory mediator production. Both live spirochetes and lipoprotein induced the transcriptional activation of several TLR pathway genes as shown in **Table 1**. The TLR1 gene transcript was significantly upregulated (*P*≤0.05) by L-OspA-stimulated BMDM from both mouse strains, and largely to the same extent. However, when the stimulant was live Bb, up-regulation of the TLR1 gene transcript in C57 BMDM was 2.6-fold that of C3H BMDM. In contrast, live Bb stimulation up-regulated the TLR2 gene transcript in C3H BMDM by 2.4-fold in relation to the C57 BMDM up-regulation and by 4.1-fold when the stimulant was L-OspA (**Table 1**). Several other genes involved in TLR signaling that were significantly expressed (*P*≤0.05) in macrophages of C57 and C3H mice include CD14, NFKBIA, TICAM2, TNFAIP3 and CEBPB. As shown in **Table 1**, the expression of some of these gene transcripts in macrophages of mice was stimulant dependent, like TNFAIP3 and CEBPB (induced more by live Bb) along with MyD88 and TICAM2 (induced more by L-OspA). Taken together, these results suggest the differential activation and expression of TLR gene transcripts in macrophages of C57 and C3H mice, with an obvious TLR2/TLR1 transcript imbalance in their macrophages.

**Table 1:** Transcription of genes related to TLR signaling is differentially induced by live spirochetes and lipoprotein in macrophages of C57 and C3H mice.

Gene group/Description	Gene	Live Bb*		L-OspA**	
Toll-Like Receptors		C57	C3H	C57	C3H
Toll-Like receptor 1	Tlr1	*3.25 (0.03)* #	*1.25 (NS)*	3.32 (0.04)	3.54 (0.01)
Toll-Like receptor 2	Tlr2	*3.49 (0.02)*	*8.10 (0.01)*	*2.80 (0.03)*	*11.44(0.04)*

Adapters & TLR Interacting Proteins					
CD14 antigen	Cd14	4.84 (0.01)	4.33 (0.02)	2.82 (0.03)	3.03 (0.03)
Myeloid differentiation primary response gene 88	Myd88	1.45 (0.01)	1.44 (NS)	**3.50 (0.01)**	**3.10 (0.01)**
Toll-like receptor adaptor molecule 2	Ticam2	1.46 (NS)	1.54 (NS)	**2.23 (0.04)**	**3.31 (0.05)**

NFkB Pathway					
Tumor necrosis factor alpha-induced protein 3	Tnfaip3	**3.55 (0.05)**	**3.87 (0.01)**	1.87 (NS)	1.30 (NS)
Nuclear factor of kappa light polypeptide gene enhancer in B-cells inhibitor, alpha	Nfkbia	3.65 (0.01)	4.02 (0.01)	4.93 (0.01)	3.41 (0.01)

NF/IL6 Pathway					
CCAAT/enhancer binding protein (C/EBP), beta	Cebpb	**3.13 (0.01)**	**5.18 (0.01)**	1.38 (NS)	1.79 (NS)

* Live Bb (live *Borrelia burgdorferi* spirochetes).

** L-OspA (lipidated outer surface protein A).

# SABiosciences RT^2^ Profiler™ PCR Array was used to quantify mRNA transcript levels of genes. Results are presented as fold increase over control (the level in unstimulated cells). *P* values shown in parentheses were obtained using the manufacturer's software; NS, not significant. *Italicized* values, differentially expressed between mice. Bolded values, differentially induced by live Bb or L-OspA.

### Exogenous IL-10 inhibits the production of inflammatory mediators produced by macrophages of C57 and C3H mice

**Figure 3:**
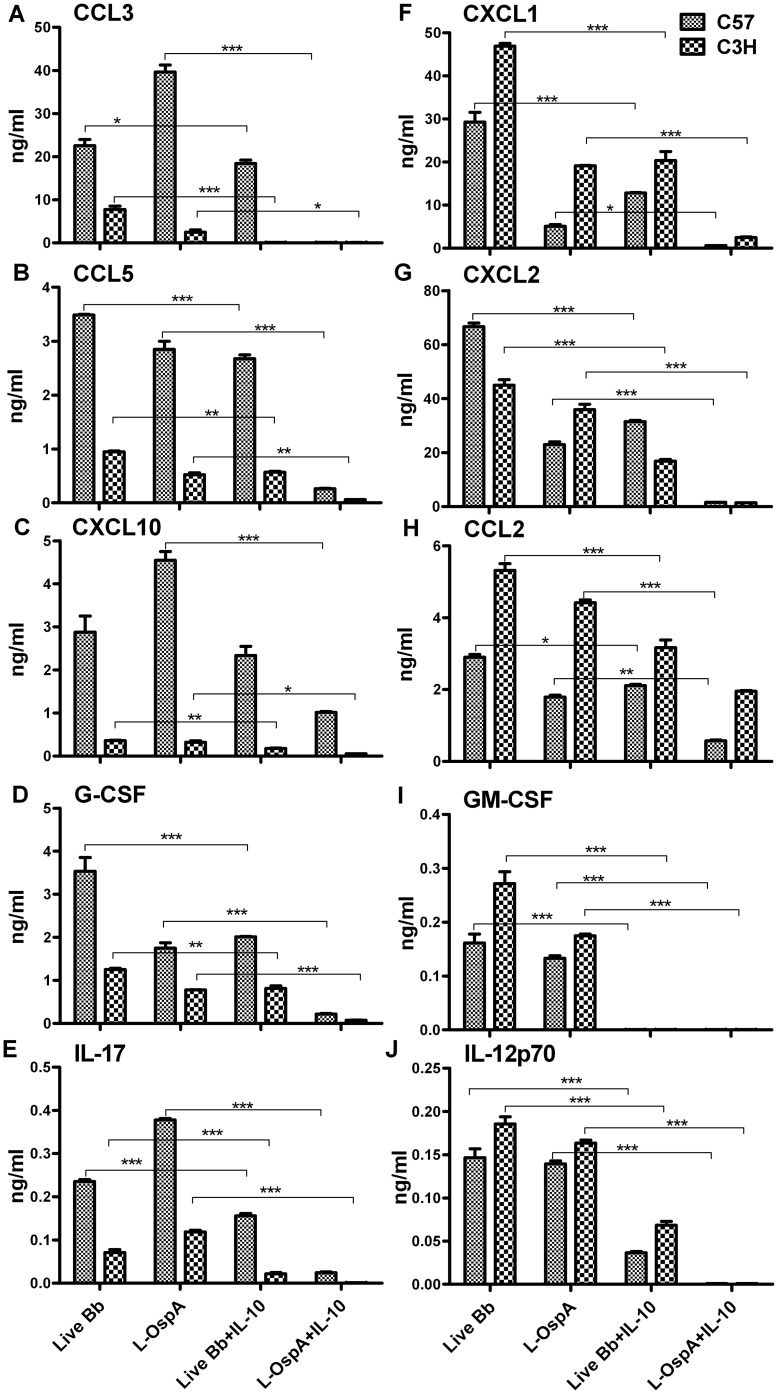
Exogenous IL-10 diminishes the levels of cytokines and chemokines, especially those induced by lipoprotein. (A–J) BMDM were stimulated and cultured as indicated in Fig. 1. In these experiments, mouse recombinant IL-10 (1 ng/mL) was added 30 min prior to addition of stimulants. Cell-free supernatants were harvested from cultures at 24 h later and protein determinations were made by multiplex ELISA. Statistical analysis was performed using one -way ANOVA followed by a Bonferroni post-hoc test along with two tailed Mann Whitney test (GraphPad Prism 5). Significance was established at *P*<0.001 = ***, *P*<0.01 = ** and *P*<0.05 = *. Each bar represents the mean ± standard deviation for duplicate cultures. Data are representative of two independent experiments, with similar results.

Previous studies using macrophages from C57 and C3H mice demonstrated that exogenous IL-10 similarly suppressed the expression levels of the prototypical pro-inflammatory mediators TNF and IL-6, as induced by the *B. burgdorferi* lipoprotein OspA [19]. We recently reported that IL-10 down-modulates genes encoding mediators of TLR signaling [27]. Our observation, reported here as well as by others [19] that BMDM of C57 mice produce higher levels of IL-10 than BMDM of C3H mice, in concert with the TLR2/TLR1 transcript imbalance we uncovered in these macrophages, prompted us to examine the effect of exogenous IL-10 on the production of inflammatory mediators. Thus BMDM were exposed to either live Bb or L-OspA in the presence or absence of recombinant mouse IL-10 at a concentration of 1 ng/mL. This was the highest IL-10 concentration achieved by BMDM from C57 mice, which is also the strain that produced the highest IL-10 levels (**Fig. 2A**). In addition, this concentration also inhibited macrophage production of TNF and IL-6, as previously reported [19]. A quantitative assessment of the effect of exogenous IL-10 on cytokines (GM-CSF, G-CSF, IL-12p70 and IL-17) and chemokines (CCL2, CCL3, CCL5, CXCL1, CXCL2, CXCL10), as produced by C57 and C3H BMDM shows the inhibitory effect of IL-10 on the production levels of these mediators (**Fig. 3A–J**). Inflammatory mediators induced by live Bb or L-OspA were similarly down-regulated by IL-10 in the two mouse strains, suggesting the ability of exogenous IL-10 to regulate inflammation in Lyme disease.

### Endogenously produced IL-10 regulates the production of inflammatory mediators, especially in macrophages from mice of the Lyme disease resistant strain

**Figure 4:**
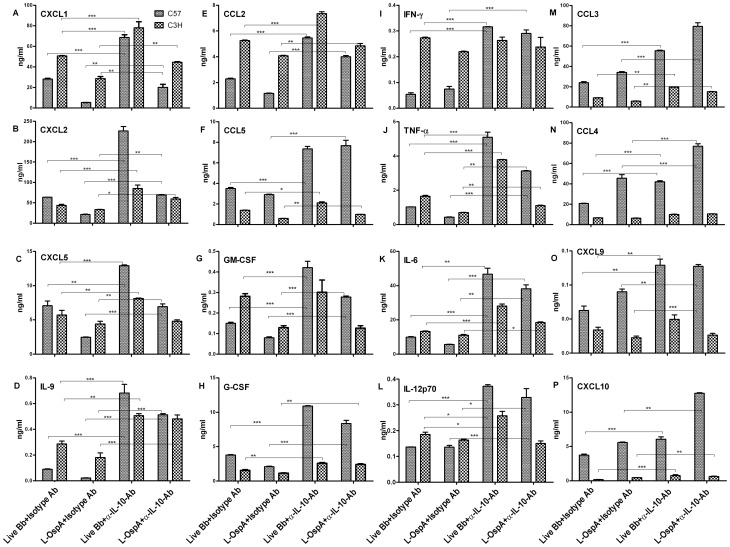
Endogenous IL-10 differentially regulates inflammatory mediators produced by macrophages of Lyme disease-resistant and -susceptible mice. (A–P) BMDM were stimulated and incubated as described in Fig. 1. For these experiments, neutralizing monoclonal antibody (Ab) against mouse IL-10 (α-IL-10) or its isotype control Ab each at 25 µg/mL were added 30 min prior to treatment with stimulants. Cell-free supernatants were harvested from cultures at 24 h and protein determinations were made by multiplex ELISA. Statistical analysis was performed as indicated in Fig. 2. Significance was established at *P*<0.001 = ***, *P*<0.01 = ** and *P*<0.05 = *. Each bar represents the mean ± standard deviation for duplicate cultures. Similar results were obtained from two independent experiments.

Given the enhanced production of IL-10 at the protein and mRNA transcript levels in BMDM of C57 mice as compared to that of C3H mice, and considering that exogenous IL-10 similarly inhibited production of inflammatory mediators in both mouse strains, we next investigated if endogenously produced IL-10 could differentially regulate the concentration levels of their respective patterns of inflammatory mediator production and particularly in the C57 mouse strain. To address this, neutralizing mouse α-IL-10 Ab at 25 µg/mL was added to cell cultures prior to the addition of either live Bb or L-OspA. This concentration was chosen based on our published studies [26]. The specificity of the effect of the α-IL-10 neutralizing Ab was determined by using a similar concentration of an isotype-matched control immunoglobulin. The concentration of IL-10 produced by BMDM in the presence of neutralizing α-IL-10 Ab in this study was below the detection limit (15.6 pg/mL and 3.2 pg/mL, respectively for single and multiplex ELISAs), suggesting complete ablation of endogenously produced IL-10. In the absence of endogenously produced IL-10 the levels of cytokines and chemokines increased in cultures of both mouse strains (**Fig. 4**). However, as evidenced in **Fig. 4A–P** neutralization of endogenously produced IL-10 significantly increased (*P*≤0.05) the concentration levels of cytokines (IL-9, IFN-γ IL-6, TNF-α, IL-12p70, G-CSF, GM-CSF) and chemokines (CCL2, CCL3, CCL4, CCL5, CXCL1, CXCL2, CXCL5, CXCL9, CXCL10) as produced by C57 BMDM whereas the corresponding increases by C3H BMDM were lower, or negligible in some cases.

### Effect of exogenous and endogenous IL-10 on fold changes in cytokine and chemokines levels as produced by BMDM from C57 and C3H mice

**Figure 5:**
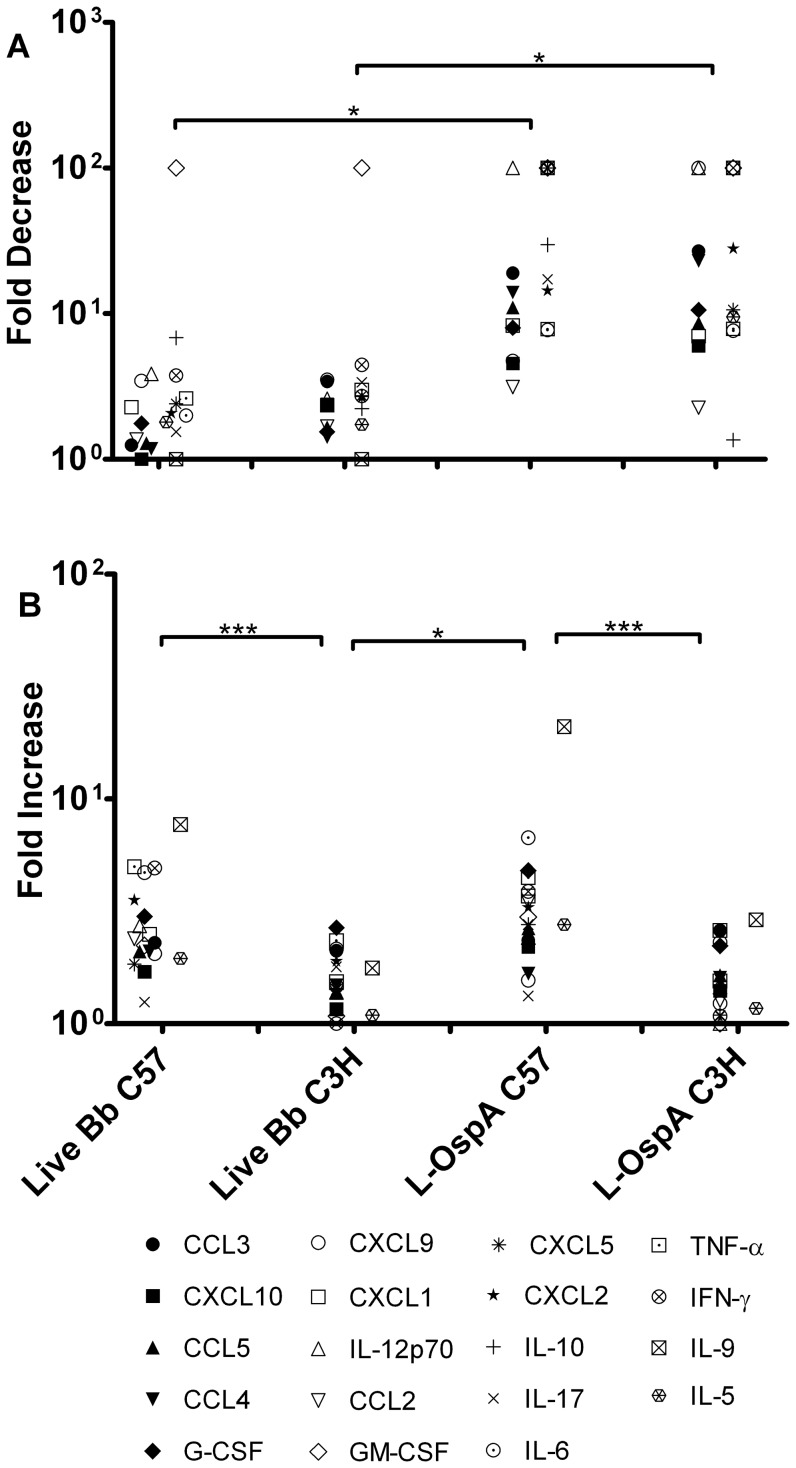
Effect of exogenous and endogenous IL-10 on fold changes in cytokine and chemokine levels as produced by macrophages from Lyme disease-resistant and -susceptible mice. BMDM were stimulated with live Bb or L-OspA in the presence or absence of exogenous or α-IL-10 (or its isotype control) Ab as described in Figs. 3 and 4. Cell-free supernatants were harvested from cultures at 24 h and protein determinations were made by multiplex ELISA. Fold decrease (A) was calculated as stimulant/stimulant exposed to exogenous IL-10. Fold increase (B) was calculated as stimulant + α IL-10 Ab/stimulant+isotype control Ab. A one-way ANOVA followed by a Bonferroni post-hoc test in conjugation with two tailed Mann Whitney test was used for data analyses. Significance was established at *P*<0.001 = ***, *P*<0.01 = ** and *P*<0.05 = *. Each symbol represents fold change for a specific cytokine or chemokine. Each fold change is representative of two independent experiments.

The results presented as fold changes in **Fig. 5** further underscore the effect of both exogenous IL-10 and removal of endogenously produced IL-10 on cytokine and chemokine production levels in live Bb- and L-OspA-stimulated BMDM cultures. The down-regulatory effect of IL-10 on inflammatory mediators was more evident when L-OspA was used as a stimulant (**Fig. 5A**), suggesting that the pro-inflammatory pathways elicited by lipoproteins are preferentially targeted by the inhibitory action of IL-10 in macrophages. In the absence of endogenously produced IL-10 the levels of cytokines and chemokines increased in cultures of both mouse strains. The effect on cytokines and chemokines secretion after removal of endogenously produced IL-10 was markedly evident in BMDM from the disease-resistant C57 mouse strain, with significantly higher fold changes for live Bb- and L-OspA-induced inflammatory mediators (*P*<0.001) (**Fig. 5B**) as compared to those of the disease-susceptible C3H mouse strain. Our observation further provides compelling evidence, and supports the notion that IL-10 is a potent regulator of inflammatory mediators in BMDM of the Lyme disease-resistant mouse strain.

### Spleen-derived macrophages recapitulate the differential pattern of cytokine and chemokine production observed in BMDM from C57 and C3H mice, as well as the effects of exogenous and endogenous IL-10

**Figure 6:**
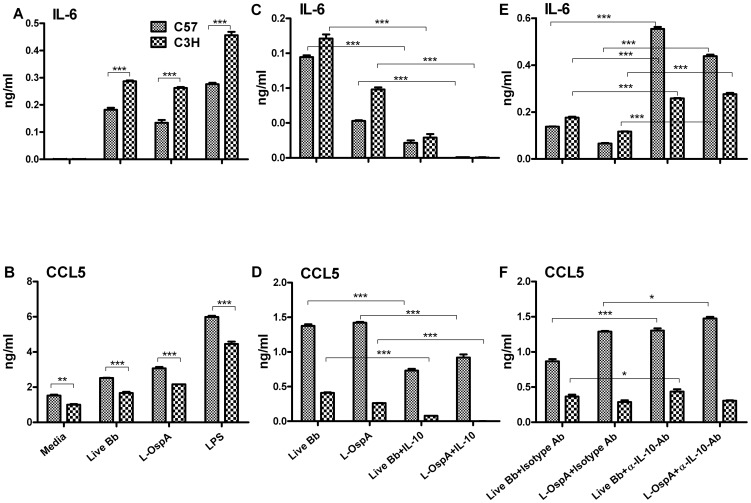
Spleen-derived macrophages recapitulate the differential pattern of cytokine and chemokine production observed in BMDM from C57 and C3H mice, as well as the effects of exogenous and endogenous IL-10. Spleen-derived macrophages (SDM) were stimulated and incubated as described in Figs. 1, 4 and 5. IL-6 and CCL5 were detected in cell-free supernatants from SDM cultures using cytokine-specific ELISAs. Statistical analysis was performed as indicated in Fig. 2. Significance was established at *P*<0.001 = ***. Each bar represents the mean ± standard deviation for duplicate cultures. These experiments were repeated two times, with similar results.

We next generated spleen-derived macrophages (SDM) from C57 and C3H mice and randomly selected one of each of the cytokines (IL-6) and chemokines (CCL5) to confirm the patterns of their BMDM-displayed inflammatory mediators. C3H SDM produced significantly higher (*P*<0.001) levels of IL-6 than did C57 SDM (**Fig. 6A**) whereas SDM from C57 mice produced higher levels (*P*<0.001) of CCL5 (**Fig. 6B**). Similar patterns of inflammatory mediators were induced when LPS was used as the stimulant (**Fig. 6A and B**). Exogenous IL-10 dampened the production levels of IL-6 and CCL5 produced by SDM of both mouse strains (**Fig. 6C and D**). Likewise, neutralization of endogenously produced IL-10 significantly (*P*<0.001) enhanced the concentrations of IL-6 (**Fig. 6E**) and CCL5 (**Fig. 6F**) to levels greater than those normally produced by SDM of both mouse strains. Removal of endogenously produced IL-10 significantly enhanced (*P*<0.001) IL-6 production by SDM of C57 to levels higher than those produced by SDM of C3H in the presence of endogenous IL-10 (**Fig. 6E**). Although we only assessed one cytokine and chemokine with SDM, the results obtained corroborate those obtained with BMDM, and provide strong support for 1) the two distinct patterns of inflammatory mediators produced by the two mouse strains, 2) exogenous IL-10 effect being directed more towards lipoprotein-induced inflammatory mediators, and 3) IL-10 controlling the production of inflammatory mediators especially in the disease-resistant mouse strain.

## Discussion

Cytokines and chemokines are thought to play key roles in Lyme disease pathogenesis. As such this study focused on understanding how qualitative and quantitative differences in inflammatory mediators production by macrophages from Lyme disease-resistant C57 and disease-susceptible C3H mice may influence disease progression. The following observations were made: (i) there are two distinct patterns of live spirochete- and lipoprotein-inducible inflammatory mediators displayed, respectively by C57 and C3H macrophages; (ii) there is a TLR2/TLR1 gene transcript imbalance in these respective macrophages; (iii) exogenous IL-10 repressed the levels of inflammatory mediators as produced by macrophages, especially when the stimulant was lipoprotein; and (iv) neutralization of endogenous IL-10 produced upon exposure of macrophages to live spirochetes or lipoprotein enhanced the concentrations of concomitantly elicited inflammatory mediators, and more so in the disease-resistant C57 than in the disease-susceptible C3H mouse strain.

The majority of cytokines and chemokines induced by live spirochetes and lipoprotein in the present study has been shown to be similarly induced in several tissues and cells from humans and mice [24], [28], [29], [30], [31], [32], [33], [34], [35], [36], [37], [38], [39]. Here we present, to our knowledge, the first report detailing two distinct quantitative patterns of inflammatory mediator production induced by either live spirochetes or lipoprotein in macrophages of C57 and C3H mice. This profile of inflammatory mediators observed in C3H macrophages is echoed by previous reports where heightened expression of TNF and IL-6 by macrophages [19], IL-1β, TNF and IL-12 in heart tissues [38] and CXCL1 and CCL2 in joints [21], [40] was seen in C3H mice. These observations suggest that the above-mentioned cytokines/chemokines may dictate, correlate with, and/or play key roles in Lyme disease-susceptibility in this mouse strain.

Correlating modified expression of specific cytokines or chemokines with resistance or susceptibility to murine Lyme arthritis has been difficult, and understandably so, since inflammatory mediators generally exert their biologic activities pluripotently and in concert with each other, either to modulate or potentiate inflammation. A case in point is the high expression of CCL2 that was reported in joints of arthritis-susceptible C3H mice, which contrasts with the finding that CCL2-receptor deficiency did not reduce arthritis severity, whereas mice deficient in KC receptor developed low-severity of Lyme arthritis [21], [40]. Nevertheless, CCL2 has been demonstrated to enhance migration of T cells across *B. burgdorferi*-stimulated HUVEC [41], and *B. burgdorferi* -specific CD4+ T cells were shown to play important roles in the severity of murine Lyme arthritis [40], [42] and carditis [43]. A second case example is the finding that arthritis development is independent of IFN-γ [24], [25], [44], [45] as verified in IFN-γ knockout mice [46], [47], yet neutralization of IL-12 in *B. burgdorferi*-infected C3H mice resulted in reduced IFN-γ expression and diminished severity of Lyme arthritis [48]. IFN-γ reportedly can promote inflammation by synergizing with *B. burgdorferi* to potentiate expression of chemokines and cytokines in human endothelial cells [49] and mouse macrophages [50] along with enhancing macrophages effector functions for spirochete clearance [51]. Together the above data illustrate that no single cytokine or chemokine may be exclusively responsible for development of spirochetal-induced pathology. Our present data further suggest that distinct quantitative patterns of inflammatory mediator production, as we found in C3H vs. C57 macrophages, may better explain susceptibility vs. resistance to Lyme disease.

In the present study, TLR1 and TLR2 genes transcripts were expressed in C57 and C3H macrophages but with an obvious TLR2/TLR1 expression imbalance in these cells. TLR2 is the putative receptor for lipoproteins [52] and *B. burgdorferi* and its lipoproteins are known to interact with TLR2/TLR1 [53] and other TLRs [54] for the release of inflammatory mediators. *B. burgdorferi* also engages receptors other than TLRs [55], [56], [57], [58] for mediating inflammatory responses, including many of the cytokines (TNF, IL-1β, IL-6) and chemokines (CCL2, CCL3, CCL5, CXCL1, CXCL2 and CXCL10) reported herein to be produced by macrophages [25], [28], [44], [55], [58]. We did not measure TLR2 or TLR1 proteins in this study but the TLR2 gene transcripts were more highly expressed by C3H macrophages after stimulation with either L-OspA or live spirochetes as compared to the expression levels by similarly stimulated C57 macrophages. These findings are in support of our previously stated hypothesis that the susceptibility to Lyme disease of the C3H mouse could be rooted in this strain's enhanced inflammatory responsiveness to lipoproteins [23]. The lower expression of the TLR2 gene transcripts in C57 macrophages may imply a TLR2 dysregulation that may have led to the engagement and/or recruitment of other receptors/co-receptors to elicit inflammatory mediators in these cells. Indeed TLR1, CD14 and MyD88 gene transcripts were highly expressed in C57 macrophages. Intergrin α_3_β_1_ was recently identified as a novel regulator that co-operates with TLR2/TLR1 in mediating two subsets of pro-inflammatory responses in human U937 macrophages stimulated with *B. burgdorferi* or lipopeptides [59]. Further studies are needed to investigate whether or not such co-operation with other receptors for example intergrin α_3_β_1_ with TLR2/TLR1 on macrophages of C57 and C3H mice, are needed to dictate their respective production patterns of live spirochete- and lipoprotein-inducible inflammatory mediators.

Our observed TLR2/TLR1 imbalance/dysregulation is not unique to the mouse model of Lyme disease. Macrophages from humans that were hypo-responsive to OspA secreted low levels of IL-6 and TNF upon stimulation with OspA, as compared with cells from normal individuals, because of an imbalance/dysregulation in TLR2/TLR1 expression on their macrophages [53]. Human PBMCs bearing TLR1 polymorphisms produced a diminished cytokine response when exposed to *B. burgdorferi* [60]. Strle and coworkers [61] also showed that TLR1 polymorphisms in some human patients triggered an exacerbated Th1 inflammatory response, especially in those with antibiotic refractory arthritis.

Exogenous IL-10 effectively modulated macrophage-produced cytokines and chemokines of both mouse strains, especially those elicited by lipoprotein. The anti-inflammatory effect of IL-10 in Lyme disease is well established both by experiments *in vitro* using various cell types and different *B. burgdorferi* stimulants [19], [20], [23], [26], [27], [62], [63] as well as *in vivo* [19], [22]. IL-10 also has been reported to prevent apoptosis of human brain endothelial cells infected with the spirochete, *Borrelia turicatae* and a lipoprotein [64]. The novelty of our present findings is that we reveal that the IL-10-mediated effect is more pronounced for lipoprotein-induced inflammatory mediators than for those induced by live spirochetes, irrespective of the mouse strain. This suggests that lipoprotein-related pathways of induction of cytokine and chemokine expression are major targets for the anti-inflammatory action of IL-10 in macrophages. At the same time the only partial IL-10 inhibitory effect on live-spirochete-induced inflammatory mediators may be due to the ability of spirochetes to also signal via TLR- [25], [28], [36], [44], [55], [56], [57], [58], [65] (and IL-10-) independent pathways. In this regard, IL-10 has been shown to inhibit chemokines in inflammatory foci depending on the stimulatory source [66]. Our results may partly explain the persistence of inflammation in tissues where there is active IL-10 synthesis during the course of Lyme disease, as recently demonstrated by the inability of adenoviral expression of IL-10 in the infected joints of C3H mice to inhibit the development of severe Lyme arthritis [22]. The use of other receptors [58], [67], [68], [69], [70], [71] and ligands [28], [72] by spirochetes to also induce inflammation is in line with evidence of *B. burgdorferi* being a master manipulator of the immune system by its ability to evade immune clearance [73], [74] or as an immunomodulator of inflammation [62], [73], [75], [76].

Macrophages from disease-resistant mice produced more IL-10 in response to stimulation with live spirochetes and lipoprotein than macrophages from disease-susceptible mice, corroborating, in part, results of previous studies [19], [74]. A number of different cell types have been reported to produce IL-10 in response to stimulation by *B. burgdorferi* or its lipoproteins [20], [26], [62], [77], [78], [79], [80], [81], [82], [83], [84]. We show here that the inhibition of the function of endogenously synthesized IL-10 by added anti-IL-10 antibody significantly enhanced the concentrations of cytokines and chemokines as produced by macrophages exposed to live spirochetes and lipoprotein, notably of the disease-resistant C57 mouse strain. Removal of endogenous IL-10 marginally impacted the level of inflammatory mediators produced by C3H macrophages indicating a lesser endogenous IL-10 mediated-effect in this mouse strain. These findings indicate the ability of IL-10 to repress inflammation in the C57 mouse, and are congruent with results by Lazarus et al. [74], where TNF and IL-6 were greatly enhanced in the B6 IL-10-/- mouse as compared to wild type controls. Others have shown that IL10-/- mice, irrespective of their genetic background, express higher levels of cytokines and chemokines in their joints than do their wild-type counterparts [24]. Furthermore, infection studies using IL10-/- mice have shown that IL-10 limits development of joint inflammation [19], [22] or IL10-/- mice are significantly better at clearing *B. burgdoferi* spirochetes at target tissue sites than are wild-type mice [74]. Sonderegger and colleagues [45] further showed the infiltration of inflammatory cytokines/chemokines and IFN-γ-producing cells in joint tissues of infected C57 IL-10 -/-, contributed to their arthritis development, substantiating IFN-γ synergy with spirochetes to promote inflammation [49], [50].

The inhibition of production of inflammatory mediators by both endogenously produced and exogenous IL-10 observed in the present study may be due to the ability of IL-10 to down-regulate transcription of multiple genes encoding mediators of the TLR pathway [27]. This interpretation is underscored by the TLR2/TLR1 imbalance in C57 macrophages, and these cells' production of high levels of inflammatory mediators following removal of endogenous IL-10. Alternatively, removal of IL-10 may have resulted in deactivation of other mediators that synergize with IL-10 to regulate inflammatory responses, such as those regulated by suppressor of cytokine signaling (SOCS) 1 and 3, whose expression is induced by live spirochetes and is enhanced in the presence of IL-10 [26]. Sahay and colleagues [31] reported that SOCS can regulate expression of cytokines produced by mouse macrophages exposed to live spirochetes- via a diminished regulation of the TLR2 pathway.

Our findings, coupled with the more effective host defense in IL-10 deficient mice, suggest that IL-10 production by innate immune cells may dictate the outcome of infection and disease severity in the mouse model of Lyme disease, especially in the disease-resistant C57 mouse, and perhaps in humans. In this regard, macrophage activation in erythema migrans lesions of patients was associated with enhanced expression of chemokines such as CXCL10, CXCL9, CXCL1 and CCL3 [85]. More recent studies provide evidence that an association may exist between clinical outcome and early, local cytokine expression in the skin of erythema migrans patients [32].

This paper unveils that the balance between resistance and susceptibility to Lyme disease correlates with, and may depend upon, a full pattern of expression of inflammatory mediators, and on the host's genetic ability to regulate such pattern. The Lyme disease-resistant C57 mouse has a genetic predisposition to a diminished innate inflammatory response with IL-10 being a key facilitator of this process. Genetic susceptibility to Lyme disease in the C3H mouse is here substantiated by its poor ability to regulate innate inflammatory responses, in part because of suboptimal endogenously produced IL-10. How IL-10 mediates its anti-inflammatory effects in macrophages of the Lyme disease-resistant and disease-susceptible mouse strains is the topic of our ongoing investigations.

## Methods

### Ethics statement

Bone marrow cells and spleens used in this study were obtained from naïve C3H/HeN and C57BL/6J mice housed at the Tulane National Primate Research Center (TNPRC). TNPRC facilities are fully accredited by the Association of Assessment and Accreditation of Laboratory Animal Care. All studies were carried out in strict accordance with the Principles for Use of Animals, the Guide for the Care and Use of Laboratory Animals, the Provisions of the Animal Welfare Act, and other applicable laws and regulations concerning the humane care and use of research animals. The protocol was approved by Tulane University's Institutional Animal Care and Use Committee (Protocol # 318). Experimental care of the animals was conducted in such a way as to minimize any discomfort or stress. Mice were sacrificed after CO_2_ inhalation; this method of euthanasia is consistent with the recommendations of the American Medical Association's Panel on Euthanasia.

### Bacteria and mouse strains

*B. burgdorferi* spirochetes (strain B31, clone 5A19, with the complete plasmid content) were grown *in vitro* in Barbour-Stoenner-Kelly (BSK)-H complete medium, as previously described [26]. Four-to-six-week-old C3H/HeN (C3H) and C57BL/6J (C57) female mice were purchased from Charles River Laboratories (Wilmington, MA). For each tissue type, cells were pooled from 5–6 mice/mouse strain/group.

### Antibodies and reagents

Purified recombinant L-OspA protein had been kindly provided by GlaxoSmithKline Biologicals (Rixensart, Belgium). The L-OspA preparation contained less than 0.25 endotoxin units per mg of protein, as assessed by *Limulus* amoebocyte assay (Associates of Cape Cod Inc., Woods Hole, MA). Neutralizing antibody (Ab) to mouse IL-10, control isotype mouse immunoglobulin (IgG1), and mouse recombinant IL-10 (rIL-10) were purchased from BD-PharMingen (San Diego, CA). Lipopolysaccharide (LPS) from *Escherichia coli* strain 026:B6 was purchased from Sigma Chemical Company (St. Louis, MO).

### Bone-marrow-derived macrophages (BMDM) and spleen-derived macrophages (SDM)

Bone-marrow cells were flushed from mouse femurs (five to six per group) with sterile Dulbecco modified Eagle medium (DMEM) containing glutamax (Gibco Invitrogen, Carlsbad, CA), washed twice and RBCs were lysed using ACK lysing reagent (Invitrogen). Cells were grown for 7 days in tissue-culture flasks at 37°C and 5% CO_2_. The culture medium consisted of DMEM glutamax supplemented with, 10% heat-inactivated fetal bovine serum (Atlanta Biological, Norcross, GA), 20 ng/mL macrophage colony-stimulating factor (M-CSF) (R&D Systems, Minneapolis, MN) and 1 µg/mL antibiotic/antimycotic (Invitrogen). The cells were harvested using TrypLE (Invitrogen) and were plated at 0.5×10^6^/well in 12-well tissue culture plates (Costar, Cambridge, MA). Mouse spleens were harvested and washed in ice-cold DMEM glutamax medium containing 10% FBS and 2× antibiotics. Tissues were triturated with sterile syringes, and the resulting cell suspensions were filtered through 40-µm nylon mesh (BD Biosciences). Spleen cells were incubated with PE-conjugated anti-F4/80 antibody (eBioscience, San Diego, CA) at 0.25 µg/10^6^ cells. The cells were washed twice, incubated with anti-PE-conjugated MACS magnetic beads, and SDM were removed by isolating positive labeled cells over magnetic MACS columns following the manufacturer's protocol (Miltenyi Biotec, Auburn, CA). Purity of SDM was greater than 95% as assessed by flow cytometry (data not shown).

### Cell cultures

The B31 5A19 spirochete culture was washed twice in antibiotic-free medium and bacteria were counted using dark-field microscopy. To determine cytokine and chemokine responses, BMDM and SDM were cultured with either 1 µg/mL of LPS or L-OspA or with live *B. burgdorferi* spirochetes (live Bb) at a multiplicity of infection (MOI) of 10∶1 for 24 h. We have shown previously that live Bb remain viable for up to 24 h in mammalian cell culture medium, as assessed by motility and by their ability to divide successfully upon transfer to BSK-H medium [27], [65]. In selected experiments, neutralizing monoclonal Ab against IL-10 (25 µg/mL) with its isotype control Ab [26], and mouse recombinant IL-10 (1 ng/mL) were used. The latter concentration was based on assessment of IL-10 production level by live spirochetes in BMDM from mice (**Fig. 2A**). Both anti-IL-10 and isotype control Abs were added to the culture medium 30 min prior to addition of the respective stimulants. All cell-free culture supernatants were collected 24 h post-stimulation by centrifugation at 450× g for 10 min, and were stored at −80°C until used.

### Measurement of inflammatory mediators

Concentrations of inflammatory mediators were quantified in cell-free supernatants of BMDM cultures using the Milliplex mouse 32-plex cytokine and chemokine detection system (Millipore Corporation, Bedford, MA) according to the manufacturer's instructions. Each sample was assayed in duplicate and cytokine/chemokine standards and quality controls supplied by manufacturer were run on each plate. The multiplex ELISA was repeated twice in two different experiments. Data were acquired on a Luminex 100 system and analyzed using bioplex manager software v4.1 (BioRad Laboratories, Hercules, CA). Multiplex test results were validated using high sensitivity Opti-EIA sets (BD-Pharmingen) or Duo sets (R&D Systems) where the latter were available. In all cases tested, comparable levels were obtained using the Luminex-based multiplex assays and individual ELISA kits (data not shown). Only those cytokine and chemokine responses that were statistically significant (*P*≤0.05) and exhibited the same pattern of production for live Bb and L-OspA in both experiments are reported in this study.

### Quantitative real-time PCR

Total RNA was isolated from BMDM treated with L-OspA or with live Bb using Trizol reagent (Invitrogen). Complementary DNA (cDNA) was produced from quality RNA (125 ng) samples using an RT^2^ first strand kit as per the manufacturer's instructions (SABiosciences, Frederick, MD). The cDNA was analyzed on a mouse Toll-like Receptor Signaling pathway RT^2^ Profiler™ PCR array (SABioscience). Ninety-six-well plates containing gene-specific primer sets for 84 relevant TLR pathway genes, housekeeping genes, and negative controls were used. After performing thermal cycling (according to the manufacturer's protocol), real-time amplification data were gathered by using ABI Prism 7900HT software. Gene expression was normalized to internal controls (housekeeping genes) to determine the fold change in gene expression between test and control samples by ΔΔCT method (SABiosciences).

### Statistical analysis

A one- or two-way Analysis of Variance (ANOVA) followed by a Bonferroni post-hoc test (GraphPad Prism 5) or two-tailed Mann Whitney test were used for cytokines and chemokines data analyses. Significance was established at *P*<0.001 = ***, *P*<0.01 = ** and *P*<0.05 = *. For qRT-PCR, significance in gene transcript levels was analyzed using the ΔΔCT method according to the manufactures' software program (SABiosciences) and significance was established at *P*<0.05.
